# Stable Cellular Senescence Is Associated with Persistent DDR Activation

**DOI:** 10.1371/journal.pone.0110969

**Published:** 2014-10-23

**Authors:** Marzia Fumagalli, Francesca Rossiello, Chiara Mondello, Fabrizio d’Adda di Fagagna

**Affiliations:** 1 IFOM Foundation - FIRC Institute of Molecular Oncology Foundation, Milan, Italy; 2 Istituto di Genetica Molecolare, CNR, Pavia, Italy; Stony Brook University, United States of America

## Abstract

The DNA damage response (DDR) is activated upon DNA damage generation to promote DNA repair and inhibit cell cycle progression in the presence of a lesion. Cellular senescence is a permanent cell cycle arrest characterized by persistent DDR activation. However, some reports suggest that DDR activation is a feature only of early cellular senescence that is then lost with time. This challenges the hypothesis that cellular senescence is caused by persistent DDR activation. To address this issue, we studied DDR activation dynamics in senescent cells. Here we show that normal human fibroblasts retain DDR markers months after replicative senescence establishment. Consistently, human fibroblasts from healthy aged donors display markers of DDR activation even three years in culture after entry into replicative cellular senescence. However, by extending our analyses to different human cell strains, we also observed an apparent DDR loss with time following entry into cellular senescence. This though correlates with the inability of these cell strains to survive in culture upon replicative or irradiation-induced cellular senescence. We propose a model to reconcile these results. Cell strains not suffering the prolonged *in vitro* culture stress retain robust DDR activation that persists for years, indicating that under physiological conditions persistent DDR is causally involved in senescence establishment and maintenance. However, cell strains unable to maintain cell viability *in vitro,* due to their inability to cope with prolonged cell culture-associated stress, show an only-apparent reduction in DDR foci which is in fact due to selective loss of the most damaged cells.

## Introduction

Upon generation of a DNA damage, cells activate a cascade of events known as DNA damage response (DDR) to coordinate the DNA repair and the transient arrest of cell-cycle progression until DNA damage has been removed in full [1]. If the DNA damage remains unrepaired, cells enter in a permanent state known as cellular senescence, associated with a persistently active DDR [2]–[5].

DDR activation has been observed upon different senescence inducing stimuli; these include genotoxic agents [5]–[12], telomere shortening or dysfunction [13]–[17] and oncogene activation [18]–[27].

Cellular senescence has been causally linked with organismal aging [28]–[31] and DDR activation has been demonstrated *in vivo* in tissues of aging mammals including primates [5], [12], [32]–[38] and in human skin naevi, which are composed of oncogene-induced senescent melanocytes [39]. DDR activity is causally involved both in the establishment and in the maintenance of cellular senescence triggered by different stimuli, as demonstrated by the loss of senescence traits upon experimental inactivation of DDR pathways [5], [11], [13], [21], [40], [41].

However, it has also been reported that while early in the senescence process DDR can be readily detected in the majority of the cells, after prolonged establishment of cellular senescence, detection of markers of DDR activation is reduced. This has led some investigators to conclude that markers of an activated DDR are detectable only in “senescing” cultures of human fibroblasts and that they are then lost when cultures are “fully senescent” [42], [43].

Therefore, it is still controversial whether cellular senescence establishment and maintenance is intrinsically and causally associated with persistent DDR activation or, instead, it is established by an initial burst of DDR signaling whose continuous activity might not be necessary for the maintenance of a senescent state. In order to address this important question, we performed long-term analyses of DDR activation in different types of human fibroblast cell strains having different characteristics and undergoing cellular senescence by different mechanisms [7], [44]–[46]. These investigations allowed us to conclude that DDR signaling is indeed maintained even for years in stable cultures of senescent cells. However, when culture conditions do not allow the long-term survival of senescent cells, DDR signaling is apparently diminished, but this is in fact due to the progressive loss of cell viability that may bias against the survival of the most damaged cells.

## Materials and Methods

### Cell Culture

Early-passage foreskin fibroblast BJ cells (The American Type Culture Collection, ATCC), lung fibroblast WI-38 cells (ATCC) and IMR-90 cells (CORIELL) were grown under standard tissue culture conditions. Replicative cellular senescence was evaluated by the failure to reach confluence after 4 weeks in culture from the last 1∶2 passage and a fraction of less than 5% of BrdU-labelled cells upon a 24-hours labelling period. Once cell population reached complete senescence, cells were kept in culture changing medium once a week and replating them once a month on new plates. BJ cells were considered young up to PD 40, pre-senescent at PD 40–60 and fully senescent at PD 80–90; WI-38 cells were considered young up to PD 30, pre-senescent at PD 35–40, fully senescent from PD 50. Cen2 and cen3, two independent batches of senescent human skin primary fibroblasts from healthy centenarians, as well as cen2tel and cen3tel cells [47], were grown under standard tissue culture conditions in DMEM supplemented with 10% fetal bovine serum, 1% L-glutamine, 1% non-essential amino acids. Senescent cen2 and cen3 cells were maintained in culture for three years changing medium every week. Oncogene-induced senescent cells (OIS BJ cells) were obtained and grown as in [21].

### Senescence associated beta-galactosidase (SA-β-gal) assay

Cells were grown on coverslips, washed in PBS, fixed in 4% paraformaldehyde for 10 minutes at room temperature, washed again and incubated at 37°C (in the absence of CO_2_) with fresh SA-β-gal stain solution containing 1 mg of 5-bromo-4-chloro-3-indolyl beta-D galactopyranoside (X-Gal) per ml, 40 mM citric acid/sodium phosphate at pH 6.0, 5 mM potassium ferrocyanide, 5 mM potassium ferricyanide, 150 mM NaCl, 2 mM MgCl_2_. Staining was evident in 2–4 hours and maximal in 12–16 hours. At the end of the incubation time, cells were washed with PBS and pictures were taken with a digital camera connected to a white light microscope. SA-β-gal activity is detected in senescent human fibroblasts as local perinuclear blue precipitate.

### BrdU staining

Cells, plated on coverslips, were incubated with 10 µg/ml BrdU (Sigma) for a 24 hours labeling period (or 60 hours for cen2 and cen3 fibroblasts); cells were fixed with 4% paraformaldheyde for 10 minutes and permeabilized with 0.2% TritonX-100 for 10 minutes at room temperature; after incubation with a blocking solution containing 2.5% BSA and 1% gelatin from cold water fish skin (SIGMA), cells were incubated with a mixture containing primary antibody (anti-BrdU 1∶20), DNase (1∶10, Stock concentration: 1 U/µl, Promega), DNase buffer and MgCl_2_ at 3 mM final concentration for 45 minutes at room temperature in a dark humidified chamber. After washing, coverslips were incubated with a secondary antibody. DAPI staining was used to detect nuclei; proliferating cells were used as a control. At least 100–300 cells (or more where indicated) were screened for a statistically significant analysis.

### Ionizing radiation

Ionizing radiation was induced by a high-voltage X-rays generator tube (Faxitron X-Ray Corporation). Cultured cells were irradiated with the indicated dose.

### Cell survival assay for IR-senescent cells

Cells were plated in 6 multi-well plates, grown until confluency and irradiated, or not, with the appropriate dose of IR. At each time point, cells were washed to remove culture medium, collected by trypsinization and counted in triplicate using a Burker chamber.

### Immunofluorescence

Cells, plated on poly-D-lysinated coverslips, were fixed and permeabilized with 1∶1 methanol/acetone solution for 2 minutes or fixed with 4% PFA for 10 minutes and permeabilized with 0.2% TritonX-100 for 10 minutes at room temperature. Cells were incubated for 30 minutes in a blocking solution containing 2.5% BSA and 1% gelatin from cold water fish skin (SIGMA) and then stained with the indicated primary antibodies for 1 hour at room temperature in a humidified chamber. Cells were washed and incubated with secondary antibodies for 1 hour at room temperature in a dark humidified chamber. DAPI staining was used to detect nuclei. At least 100–300 cells were screened for a statistically significant analysis.

### Immunoblotting

Cells were lysed in sample buffer and 50 µg of whole cell lysate were resolved by SDS-PAGE, transferred to nitrocellulose and probed as in [21].

### Antibodies

Anti-γH2AX (Millipore, 05–636, 1∶200); anti-ATM pS1981 (Rockland, 200-301-400, 1∶400); anti-pS/TQ (Cell Signalling, 2851, 1∶200); anti-53BP1 (mouse, gift from T. Halazonetis, University of Geneva, 1∶20; rabbit, Novus NB100-304, 1∶200); anti-BrdU (Becton Dickinson, 347580, 1∶20); anti-p16 (H-156) (Santa Cruz Biotechnologies, 1∶500 for immunoblotting and 1∶200 for immunofluorescence); anti-vinculin clone hVIN-1 (SIGMA, 1∶5000).

### Statistical analysis

Results are shown as means or percentages plus or minus standard error of the mean (s.e.m.) or standard deviation (s.d.) as indicated; P value was calculated by Student's two-tailed t-test or chi-squared test, respectively.

## Results

### DDR signaling persists for years after cellular senescence establishment

**Figure 1:**
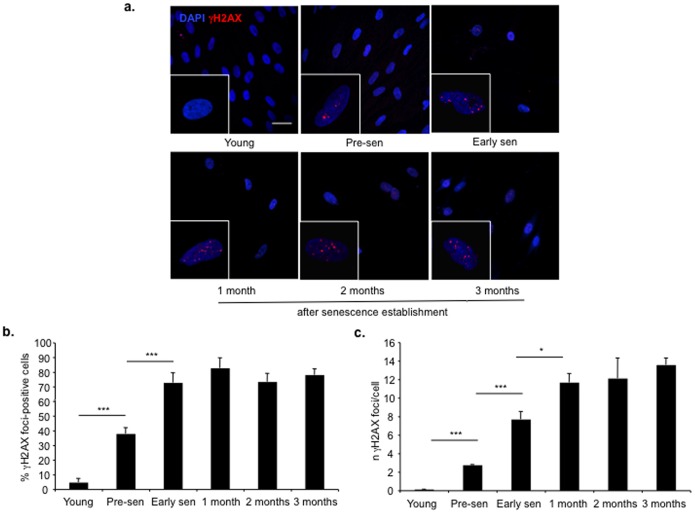
DDR is detectable months after establishment of telomere-initiated cellular senescence. **a.** In BJ cells, DDR, in the form of γH2AX foci, is detectable long time (up to three months) after establishment of telomere-initiated cellular senescence. Scale bar, 40 µm. **b.** Bar graph shows the fraction of γH2AX foci-positive cells ± s.e.m. before (young and pre-sen) and at the indicated time points after senescence establishment. Cells were considered positive if bearing more than 3 DDR foci (*** p-value <0.001). **c.** Bar graph shows the average number of γH2AX foci ± s.e.m. per cell at the indicated time points before and after senescence establishment (*** p-value <0.001; * p-value <0.05).

We analyzed human foreskin fibroblasts, BJ cells, immediately following their entry into replicative senescence state; BJ cells have been demonstrated to undergo cellular senescence because of telomeres shortening, since introduction of telomerase (hTERT) allows senescence bypass and immortalization [48]. We tested if, once cellular senescence is established, DDR is persistently associated with this condition or it is eventually switched off. To this aim, the presence of DDR foci was followed over time at the single-cell level by immunofluorescence, before and at different time points following senescence establishment (1, 2, 3 months). We consistently detected DDR foci containing γH2AX, ATM pS1981 and pS/TQ signals, throughout the different time points studied, even after 3 months, the last time point studied (Figs. 1 and S1). Therefore, we conclude that DDR is persistently active even months after entry into cellular senescence.

**Figure 2:**
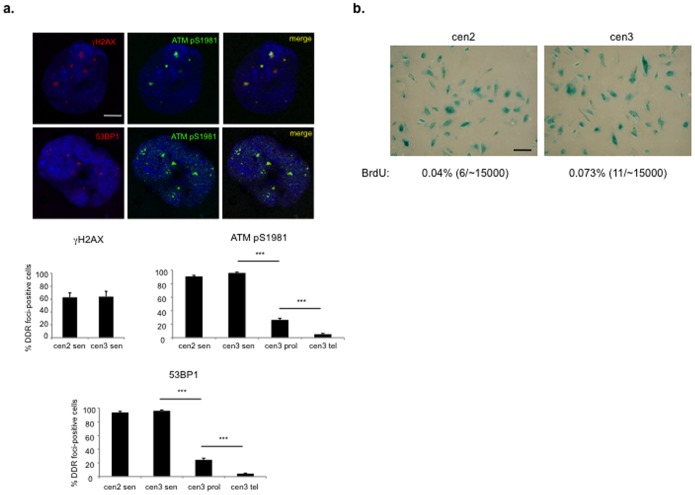
Telomere-initiated senescent cells retain active DDR foci for years after senescence establishment. **a.** DDR, in the form of ATM pS1981 foci co-localizing with 53BP1 and γH2AX foci, is detectable three years after senescence establishment. Scale bar, 10 µm. Below, bar graphs show the percentage of cells positive ± s.e.m. for the indicated DDR markers, in senescent (sen), early passage proliferating (prol) or telomerized proliferating (tel) skin fibroblasts from two independent centenarian donors (cen2 and cen3). Cells were considered positive if bearing more than 3 DDR foci (*** p-value <0.001). **b.** SA-β-gal staining of the two batches, cen2 and cen3, is shown together with the percentage of BrdU-positive cells. Scale bar, 100 µm.

Cellular senescence can be a very stable condition and anecdotic evidence indicates that senescent cells can be maintained in culture for years. To extend our observations, we searched for and gained access to two independent batches of human skin fibroblasts, named cen2 and cen3, which had undergone telomere-initiated replicative senescence three years before our analysis [47] and have been kept in culture under standard tissue culture conditions since then. As expected, virtually all cells stained strongly positive for senescence-associated beta-galactosidase (SA-β-gal) activity, a marker of cellular senescence and a prolonged (60 hours) pulse of BrdU revealed only 6–11 BrdU-positive nuclei among 15000 plated cen2 or cen3 cells, indicating that less than 0.08% of cells were still proliferating (Fig. 2b). Thus, these cells in cultures can be considered very deeply senescent.

When we analyzed these cultures for the presence of DDR foci, we discovered that the vast majority (70–80%) of cells in both cultures still displayed markers of DDR activation containing ATM pS1981, co-localizing with 53BP1 and γH2AX; their morphology and number per cell was similar to that previously observed in senescent cultures (Fig. 2a). For comparison, the plots in figure 2a show the low percentage of DDR positive cells in early passage proliferating cells and of telomerized proliferating cells (∼25% and ∼5% respectively) analyzed in parallel. Therefore, DDR can be persistently active even years after senescence establishment.

In summary, these results show that a DDR is triggered at the onset of cellular senescence and that its activity persists in time in senescent cells for months and years, likely indefinitely.

### DDR loss in senescent cultures is associated with cells loss

Our observation, although supported by several studies [11], [13], [14], [17], [32], [34], [40] is in contrast with other reports, which describe a progressive loss of DDR signaling following full establishment of senescence in culture [42], [43].

In order to reconcile these apparently conflicting results, we decided to extend the long-term analysis of DDR to another type of human fibroblasts, WI-38, a cell strain deriving from fetal lung. This cell strain has some features distinct from BJ cells [44]: besides the different origin, WI-38 cells have different antioxidant properties, with BJ cells having higher antioxidant capacity. Importantly, WI-38 cells senesce not because of telomere shortening like BJ cells, but rather because of their inability to cope with the stress induced by standard culture conditions and 20% atmospheric oxygen. This is consistent with the inability of telomerase to immortalize them [44], or prevent stress-induced cellular senescence [7], although in certain conditions WI-38 cells can escape the senescence-associated cell cycle arrest, acquiring potentially malignant alterations [49]. Another evidence of the higher sensitivity of WI-38 to stress conditions is also shown by the activation of the p16/pRb pathway (Fig. S2 and [45]) that has been observed associated with growth arrest in cells grown under inadequate conditions, such as exposure to oxidative stress or strong mitogenic stimuli [50]–[52].

**Figure 3:**
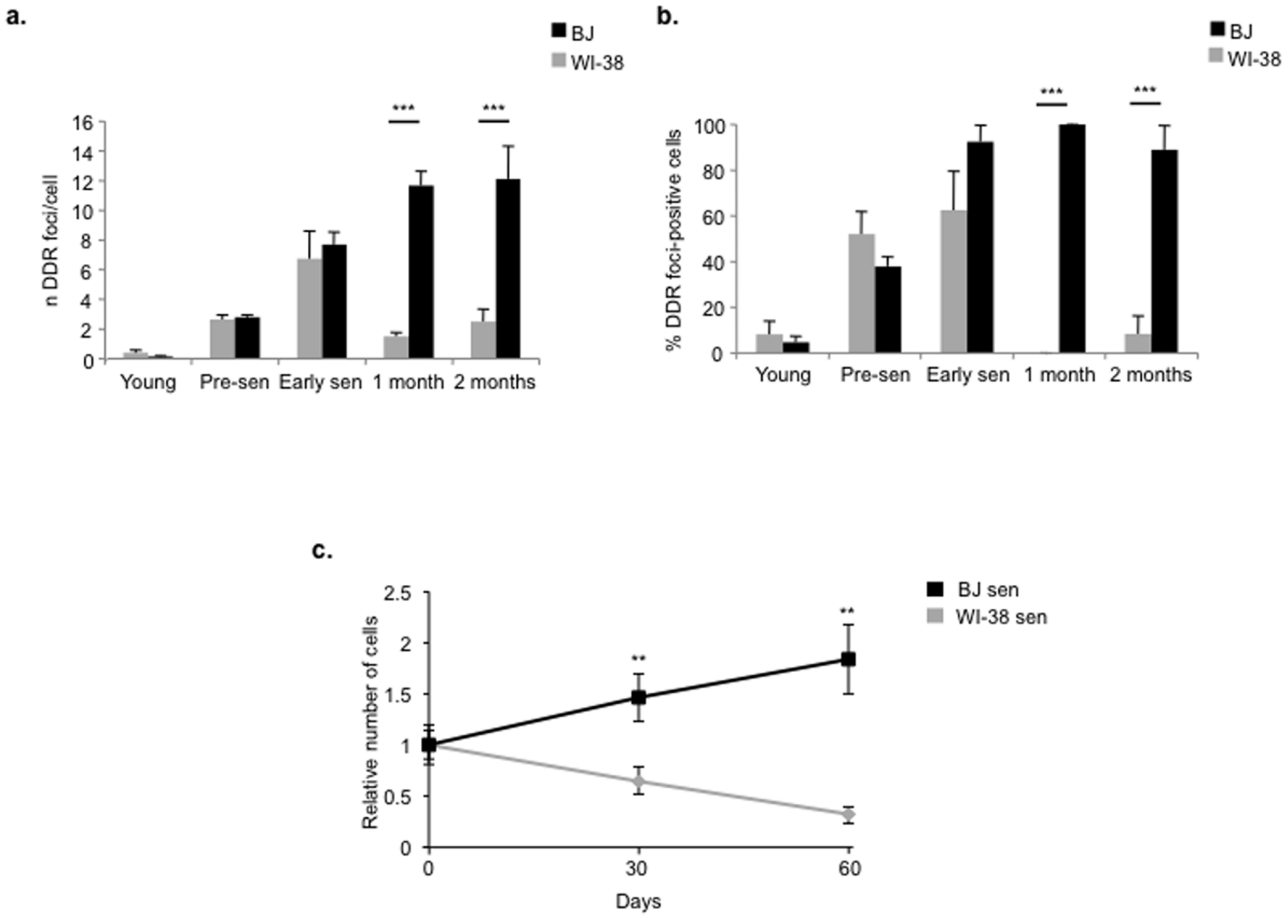
Unstable cellular senescent state is associated with loss of DDR foci. **a.** Bar graphs show the average number of γH2AX foci ± s.e.m. per cell before (young and pre-sen) and at the indicated time points after senescence establishment in two different types of fibroblast cell strains, BJ and WI-38. **b.** Bar graphs show the fraction of γH2AX foci-positive cells ± s.e.m. at the indicated time points after senescence establishment in BJ *vs* WI-38. Cells were considered foci-positive if the number of foci were more than three (*** p-value <0.001). **c.** WI-38 senescent cells (WI-38 sen) in culture tend to reduce in number with time (day 30 and day 60) whereas BJ senescent cells (BJ sen) do not. The graph shows the fold change in cell number ± s.d. normalized to the number of senescent cells plated at day 0 (** p-value <0.01).

We analyzed WI-38 fibroblasts before and as they entered replicative senescence, as assessed by BrdU incorporation and SA-β-gal staining (Fig. S3). Also WI-38 fibroblasts show evidence of DDR activation when they enter into senescence, with γH2AX foci co-localizing with other members of the DDR, such as 53BP1 and pS/TQ (Figs. 3a, b, S4 and data not shown), while DDR foci are absent in early passage cells. We thus tested the stability of DDR after replicative senescence establishment in this cell strain; we compared BJ and WI-38 cells before, immediately and 1 month or 2 months after senescence establishment by monitoring DDR signaling in the form of γH2AX foci. We observed that the fraction of DDR-positive WI-38 senescent cells decreases with time in culture. This is different from BJ cells that show an increased percentage of DDR-positive cells that remains stable both in early and in late senescence, as shown in Figs. 3a, b, S4 and S5. Importantly, one-month old WI-38 senescent cells, with a lower number of DDR-foci, were still able to mount an active DDR, as detected in the form of γH2AX foci, upon acute DNA damage generation following exposure to 20 Gy X-rays (Fig. S6): this suggests that WI-38 senescent cells still retain the ability to activate DDR upon generation of DNA damage and the decrease in the DDR foci is not due to a progressive impairment of the ability to activate DDR signaling in these cells.

However, we also counted the number of WI-38 or BJ senescent cells, at different time points (day 30 and day 60 after plating) and compared them to their initial number (day 0). We noticed that, despite identical growth conditions, the number of WI-38 senescent cells in culture decreases in time, whereas BJ senescent cells demonstrated a more stable condition and their number did not decrease (Fig. 3c) – the apparent small increase in number of BJ cells is consistent with the small number of BrdU-positive cells detected in senescent cultures and indicates a doubling time longer than 60 days.

These results indicate that upon replicative cellular senescence, DDR is robustly activated in both cell types. However, senescent WI-38 cells tend to die or be lost from the cultures with time, likely as a result of their inability to cope with standard culture conditions. As a consequence, it is not possible to draw a firm conclusion on DDR stability under these conditions since loss of cells may not be random and this makes the result not unambiguously interpretable. Indeed, it is likely that cells carrying more damage will be preferentially lost, thus skewing the analysis toward the remaining less damaged and thus less DDR-positive cells.

**Figure 4:**
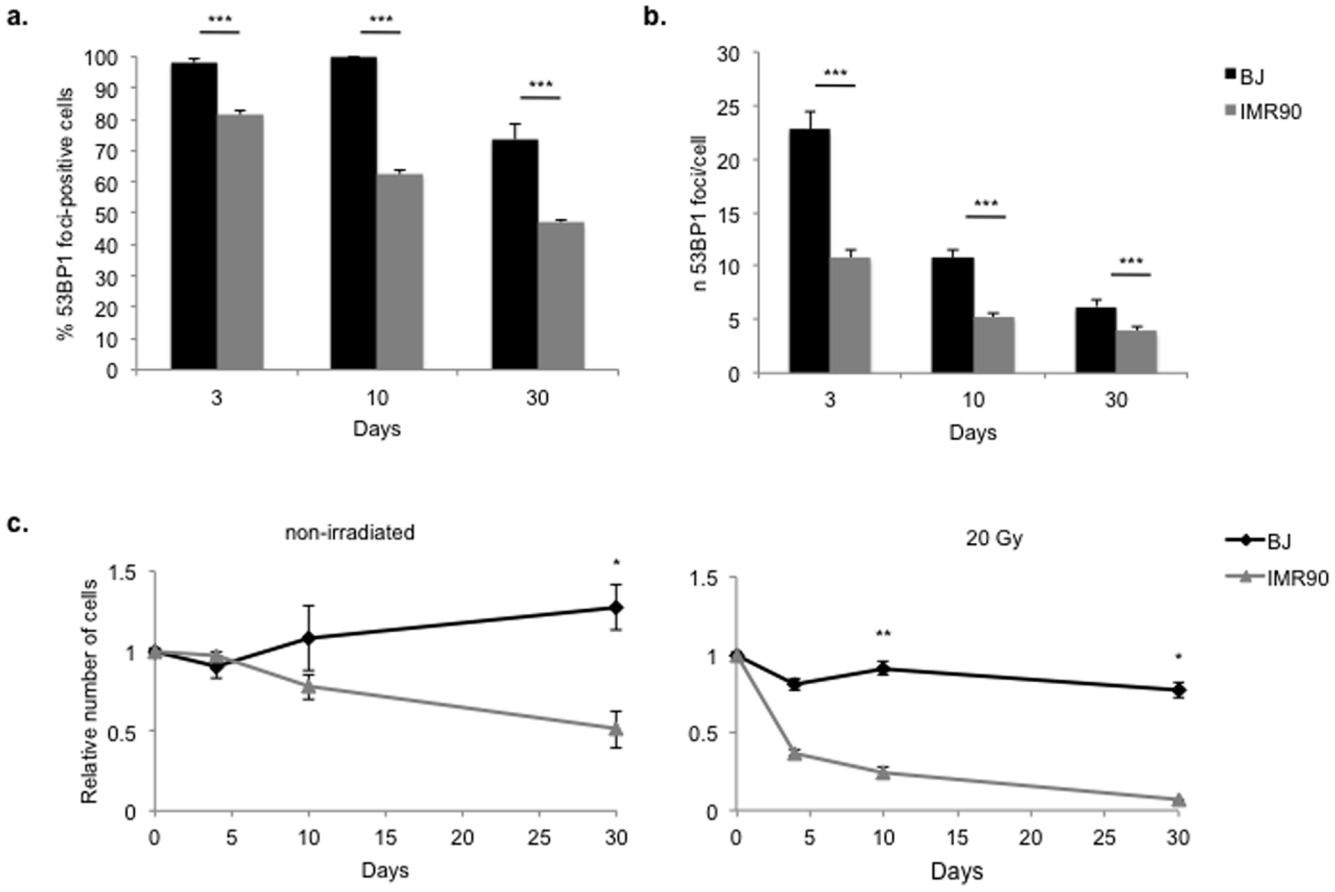
Apparent differential DNA damage response activation during IR-induced cellular senescence correlates with cell survival. **a–b.** BJ and IMR-90 cells were irradiated with 20 Gy and stained for 53BP1, as a DDR marker, three, ten and thirty days later. Bar graphs show the quantification of 53BP1-positive cells and number of 53BP1 foci per cell ± s.e.m. Cells were considered positive if bearing more than 3 DDR foci (*** p-value <0.001). **c.** BJ and IMR-90 cells were irradiated or not with 20 Gy. Graphs show the average cell number ± s.d. at different time points, in irradiated cells and non-irradiated controls (* p-value <0.05; ** p-value <0.01).

DNA damage-induced cellular senescence, caused by ionizing radiations (IR) or chemotherapeutic drugs, has been shown to be caused by persistent DDR activation fuelled by irreparable DNA damage [5], [12]. We therefore extended our analyses to IR-induced cellular senescence in two different human fibroblasts cell strains. Like WI-38, also IMR-90 are sensitive to cell culture stress, as only culture at 3% oxygen or antioxidant supplemented medium, but not telomerase expression alone, is needed to immortalize them [46], although this has not always been observed [53]. We irradiated early passage, contact-inhibited BJ and IMR-90 cells and stained them for 53BP1 as a DDR marker. These cells showed a comparable number of DDR foci one hour after low dose irradiation (Fig. S7). However, at later time points (3, 10 and 30 days) post 20 Gy irradiation, IMR-90 showed a reproducibly lower number of 53BP1 foci per cell and lower fractions of 53BP1-positive cells at various time points (Figs. 4a, b, S8 and S9). This further suggests that the presence of persistent DDR foci upon senescence establishment can apparently vary among cell strains. As suggested above for replicative senescence, this different behaviour could be explained by the differential sensitivity to stress of different cell types. To test this, we monitored cell survival after exposure of the two cell strains to high doses of IR: we observed a massive induction of cell death in IMR-90 upon irradiation and, to a minor extent, also in non-irradiated cells. Differently, the number of BJ cells remained fairly constant (Fig. 4c).

In summary, these results indicate that DDR signaling persists in senescent cells, provided that their viability in culture is maintained.

## Discussion

Although a causative role of DDR signaling is generally accepted in the establishment of cellular senescence, its role in senescence maintenance, meaning after many days following its establishment, has been put into question [42], [43]. The observations here reported allow to reconcile the seemingly contradictory results on the role of DDR in the maintenance of established cellular senescence. We propose that cellular senescence is a stable and permanent condition and as such it is stably associated with a prolonged, or permanent, DDR. This is based on our results here reported on different cell strains studied up to three years post senescence establishment. This is however provided that cells remain viable in culture for prolonged times and no cell death and consequent loss of cells from the culture occurs. Although alternative interpretations of these results can be imagined, we propose that if suboptimal growth conditions and/or an intrinsic frailty of some cell strains causes a loss of cells during their prolonged culture *in vitro*, as evinced by declining numbers of adherent cells in culture, this is associated with a reduction of detectable DDR-positive senescent cells. It is likely that in this process the most damaged (and therefore most DDR-positive) ones are lost and this explains the, only apparent, progressive reduction of DDR signaling in these senescent cultures.

The mechanism of cell loss observed in replicative senescent WI-38 and IR-induced senescent IMR-90 is presently unclear. We did not observe an evident induction of apoptosis, as detected by caspase 3 cleavage, or necrosis as monitored by propidium iodide positivity (data not shown). However, this can also be explained by the fact that these events occur over a considerable length of time, making them hard to detect.

It has been proposed that the permanent cell cycle arrest associated with cellular senescence is a tumor suppressive mechanism [21], [25], [39], [54]–[56]. Presently two main mechanisms have been invoked to explain the permanent arrest of senescent cells: the activation of DDR signaling pathways enforcing a chronic DNA damage-induced checkpoint arresting cell-cycle progression, and the establishment of heterochromatin formation in the form of senescence-associated heterochromatin foci (SAHF) [57] which have been proposed to suppress the transcription of E2F-target genes, necessary to drive cell proliferation. We have previously reported that the establishment and maintenance of the senescent state can occur in the absence of SAHF and heterochromatin formation [58]. Indeed, cellular senescence can be observed in the absence of SAHF and, conversely, SAHF-like structures can be detected in proliferating cells that have bypassed senescence [58]. Therefore, the available evidence indicates that DDR signaling is the main pathway responsible for DDR activation. The observation that DDR can be detectable in senescent melanocytes within naevi, that have been likely senescent for years or months [39], further strengthens the conclusion that DDR is stably associated with the cellular senescence condition.

## Supporting Information

Figure S1 **DDR is detectable months after establishment of telomere-initiated cellular senescence.** **a.** Representative pictures shows that DDR, in the form of ATM pS1981 (red) or pS/TQ (green) foci (merge in yellow), is still detectable three months after senescence establishment in BJ cells. Scale bar, 10 µm. Percentages in the picture show the fraction of ATM pS1981 or pS/TQ foci-positive cells ± s.e.m. **b.** Bar graphs show the average number of ATM pS1981 or pS/TQ foci ± s.e.m. per cell at the indicated time points (*** p-value <0.001).
(PPTX)

Figure S2 **Replicative senescence in WI-38 cells is associated with p16 activation.**
p16 protein level detection by immunofluorescence (a) or by immunoblotting (b) shows an increase in WI-38 senescent cells but not in BJ senescent cells. Scale bar, 100 µm. In b, proliferating BJ (BJ prol) and WI-38 (WI-38 prol) cells are shown as negative control; OIS BJ cells were used as positive control for p16 accumulation. Vinculin was used as a loading control.
(PPTX)

Figure S3 **Senescence establishment in WI-38 and BJ fibroblasts detected by SA-β-gal staining and BrdU incorporation assay.** WI-38 and BJ senescent cells (WI-38 sen and BJ sen respectively) show low level of BrdU incorporation (24 hours pulse) compared to the pre-senescent (pre-sen) and proliferating ones (prol) and an increase in SA-β-gal activity. Error bars represent s.e.m.
(PPTX)

Figure S4 **Replicative senescent WI-38 cells tend to lose DDR activity with time.** Representative pictures show DDR foci in the form of γH2AX followed over time, up to 1 month from the entry into senescence (early senescent cells); whereas senescent BJ maintain the DDR, WI-38 tend to lose it with time. Scale bar, 5 µm.
(PPTX)

Figure S5 **DDR foci distribution in BJ and WI-38 before and at different time points after senescence establishment.** Histograms show the distribution of γH2AX foci for data in Fig. 3a.
(PPTX)

Figure S6 **Both senescent WI-38 and BJ cells can mount a proficient DDR upon acute DNA damage.** WI-38 senescent cells (WI-38 sen) are still able to mount a DDR as detected in the form of γH2AX foci 1 day after induction of DNA damage by irradiation with 20 Gy, similarly to BJ senescent cells (BJ sen). Scale bar, 5 µm.
(PPTX)

Figure S7 **BJ and IMR-90 activate a comparable DDR initially upon irradiation.** BJ and IMR-90 cells were irradiated with 1 Gy and stained 10 minutes later. **a.** Representative images of 53BP1 foci. Scale bar, 20 µm. **b.** Quantification of number of 53BP1 foci per cell.
(PPTX)

Figure S8 **Differential kinetics of DDR foci resolution in different cell types.** Representative pictures of 53BP1 foci in BJ and IMR-90 at the indicated time points after 20 Gy irradiation. Scale bar, 20 µm.
(PPTX)

Figure S9 **DDR foci distribution in BJ and IMR-90 at different time points following irradiation.** Histograms show the distribution of 53BP1 foci for data in Fig. 4b.
(PPTX)
