# Supplementary material for: Stable Cellular Senescence Is Associated with Persistent DDR Activation
Source: PLoS One. 2014 Oct 23;9(10):e110969. doi: 10.1371/journal.pone.0110969 (PMC4207795; doi:10.1371/journal.pone.0110969)
Supplement: Figure S1 — DDR is detectable months after establishment of telomere-initiated cellular senescence. a. Representative pictures shows that DDR, in the form of ATM pS1981 (red) or pS/TQ (green) foci (merge in yellow), is still detectable three months after senescence establishment in BJ cells. Scale bar, 10 µm. Percentages in the picture show the fraction of ATM pS1981 or pS/TQ foci-positive cells ± s.e.m. b. Bar graphs show the average number of ATM pS1981 or pS/TQ foci ± s.e.m. per cell at the indicated time points (*** p-value <0.001). (PPTX) [file pone.0110969.s001.pptx]

## Slide 1
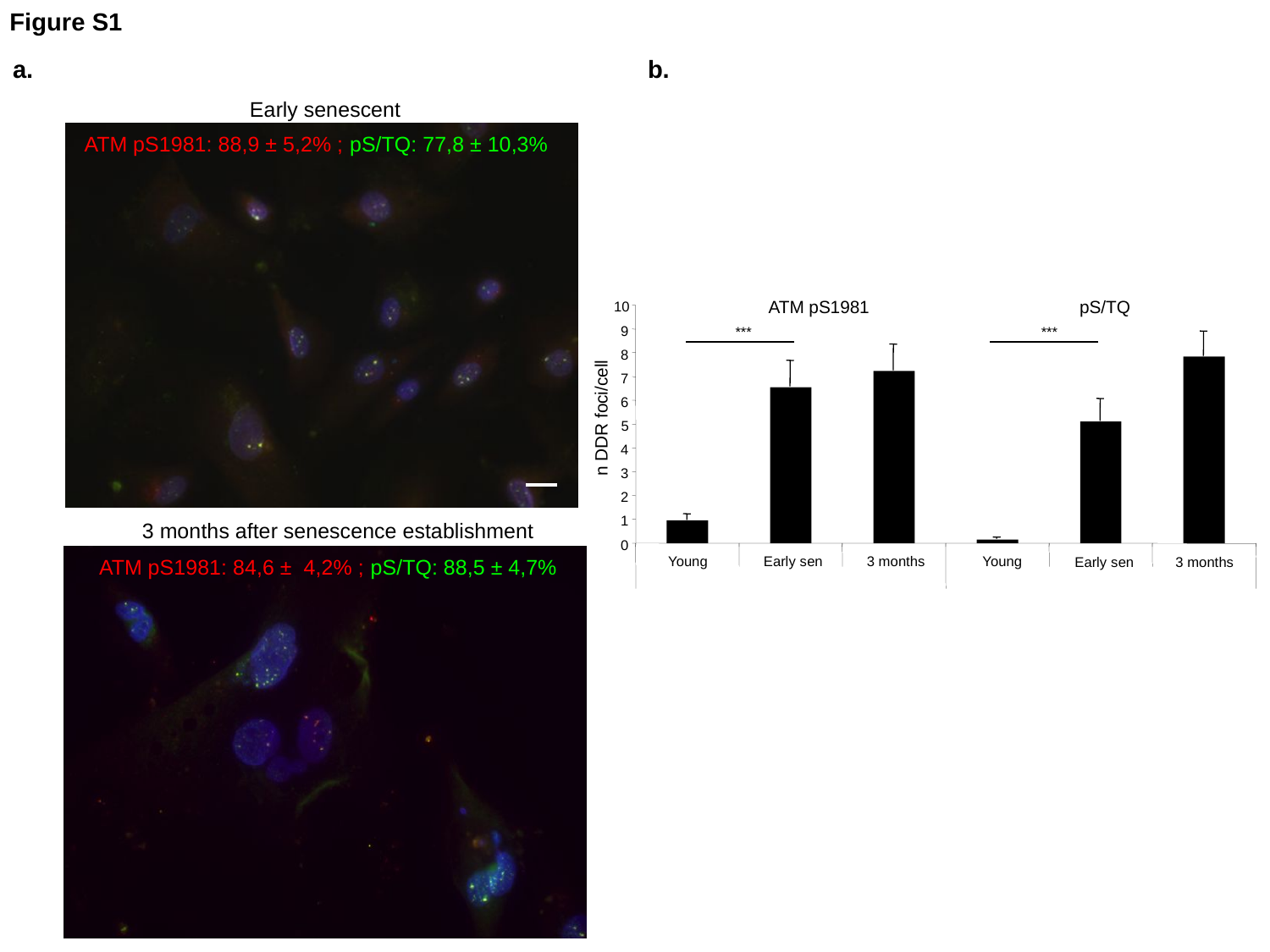

Figure S1
a.
b.
Early senescent
ATM pS1981: 88,9 ± 5,2% ; pS/TQ: 77,8 ± 10,3%
ATM pS1981
pS/TQ
10
9
***
***
8
7
6
n DDR foci/cell
5
4
3
2
3 months after senescence establishment
1
0
ATM pS1981: 84,6 ± 4,2% ; pS/TQ: 88,5 ± 4,7%
Young
Early sen
3 months
Young
Early sen
3 months
