# Supplementary figures and images for: Stable Cellular Senescence Is Associated with Persistent DDR Activation
Source: PLoS One. 2014 Oct 23;9(10):e110969. doi: 10.1371/journal.pone.0110969 (PMC4207795; doi:10.1371/journal.pone.0110969)

## Slide 1
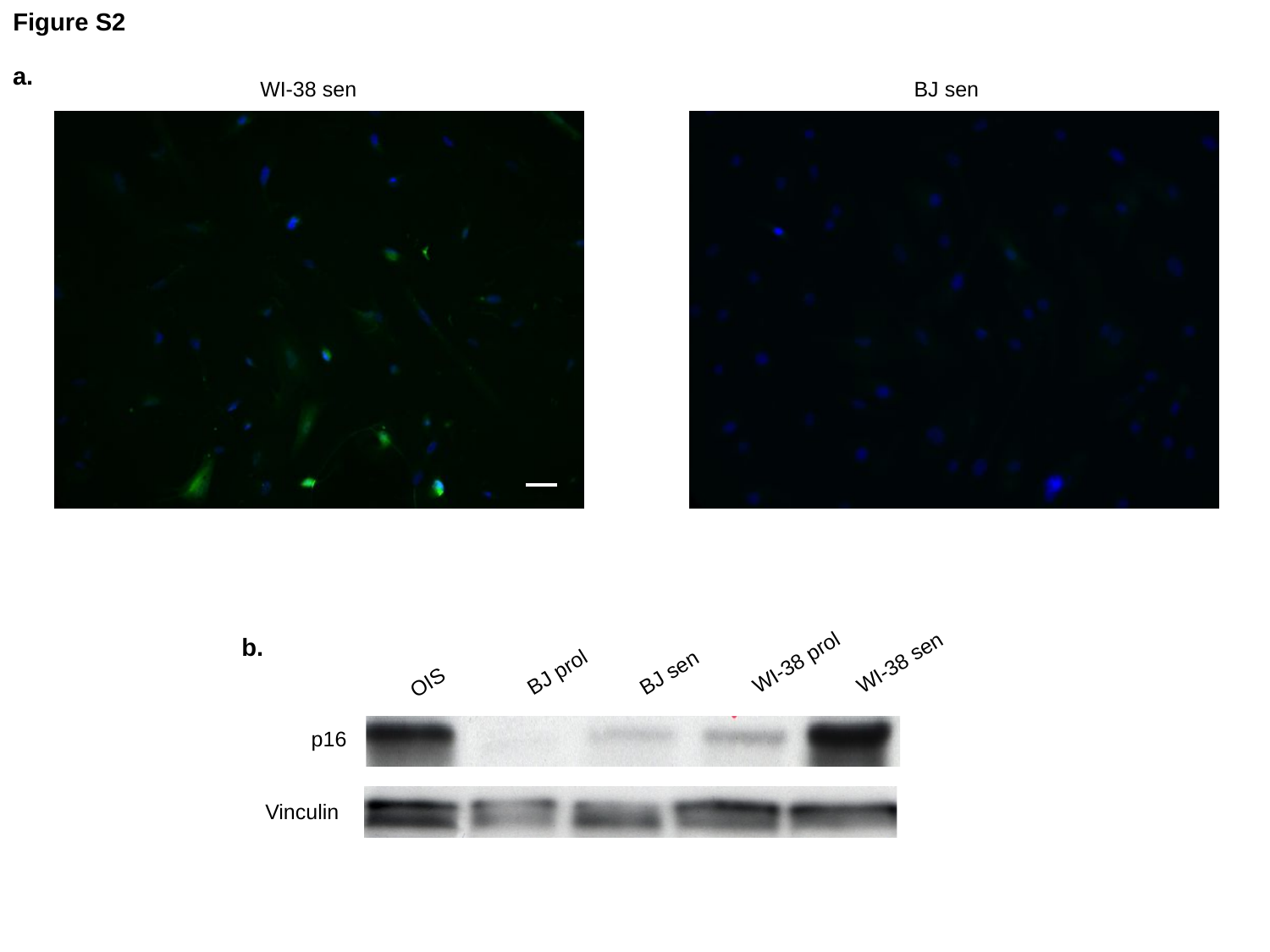

Figure S2
a.
WI-38 sen
BJ sen
b.
WI-38 prol
WI-38 sen
BJ prol
BJ sen
OIS
p16
Vinculin

Supplement: Figure S2 — Replicative senescence in WI-38 cells is associated with p16 activation. p16 protein level detection by immunofluorescence (a) or by immunoblotting (b) shows an increase in WI-38 senescent cells but not in BJ senescent cells. Scale bar, 100 µm. In b, proliferating BJ (BJ prol) and WI-38 (WI-38 prol) cells are shown as negative control; OIS BJ cells were used as positive control for p16 accumulation. Vinculin was used as a loading control. (PPTX) [file pone.0110969.s002.pptx]

## Slide 1
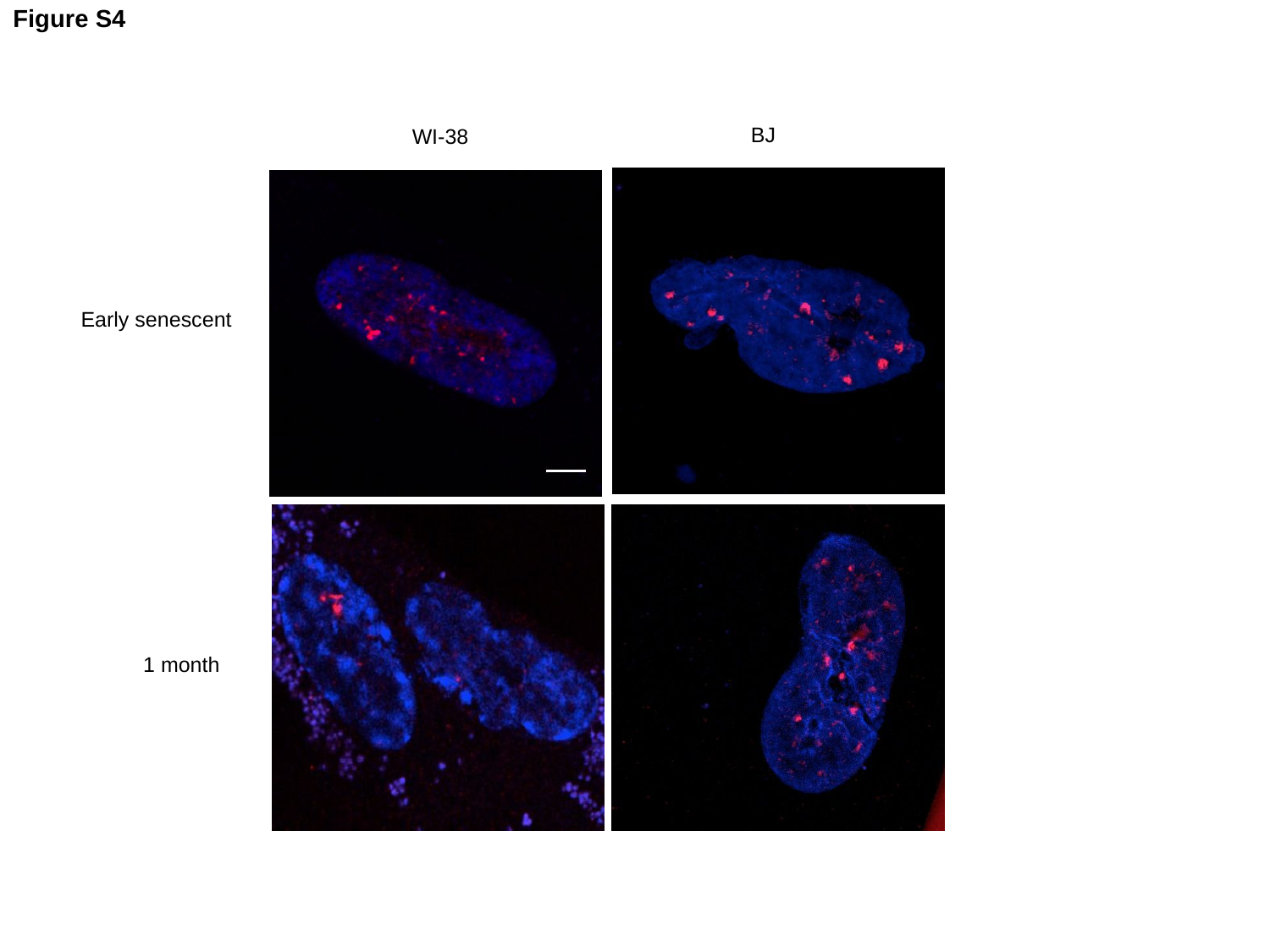

Figure S4
BJ
WI-38
γH2AX
Early senescent
1 month

Supplement: Figure S4 — Replicative senescent WI-38 cells tend to lose DDR activity with time. Representative pictures show DDR foci in the form of γH2AX followed over time, up to 1 month from the entry into senescence (early senescent cells); whereas senescent BJ maintain the DDR, WI-38 tend to lose it with time. Scale bar, 5 µm. (PPTX) [file pone.0110969.s004.pptx]

## Slide 1
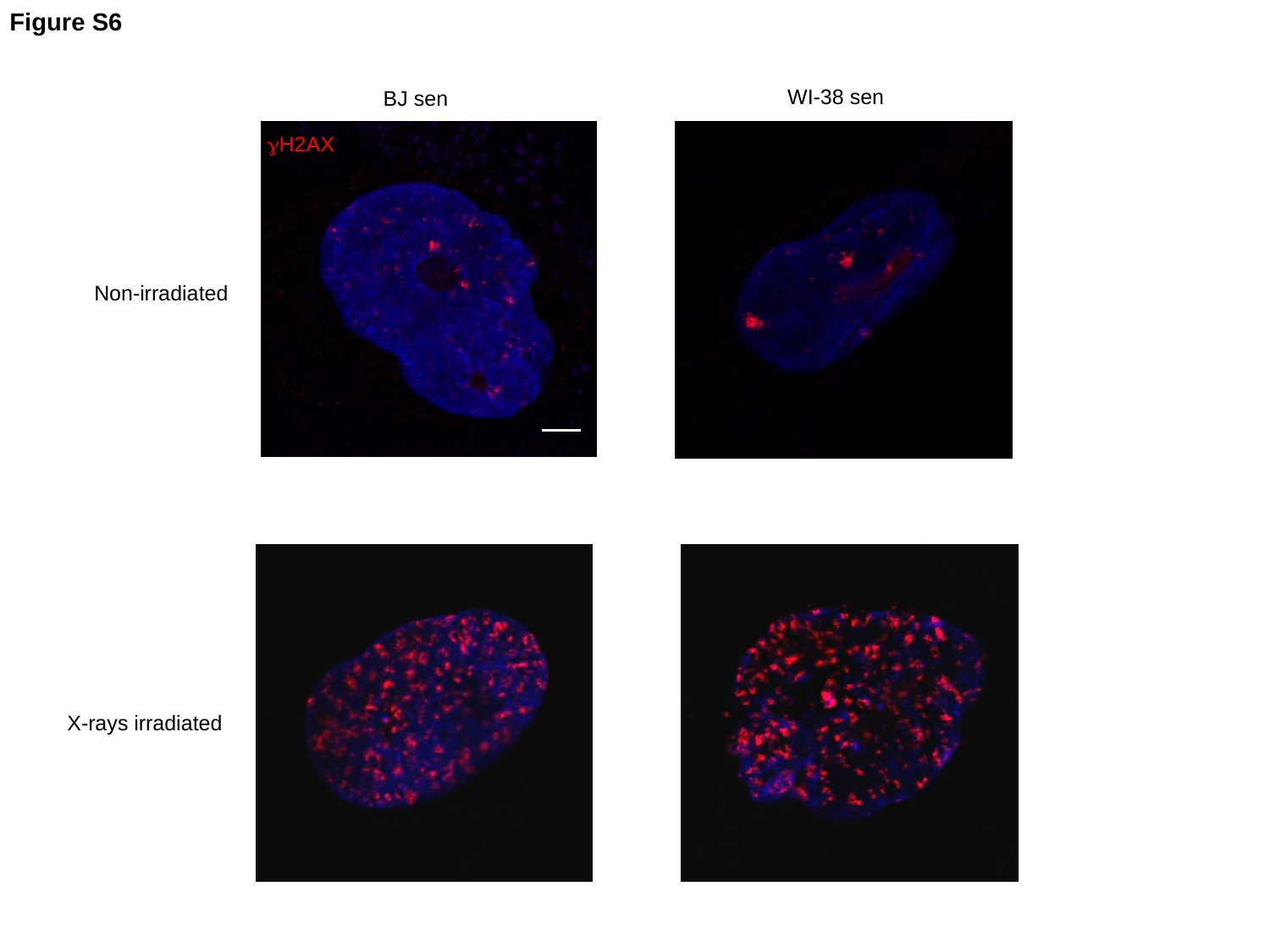

Figure S6
WI-38 sen
BJ sen
γH2AX
Non-irradiated
X-rays irradiated

Supplement: Figure S6 — Both senescent WI-38 and BJ cells can mount a proficient DDR upon acute DNA damage. WI-38 senescent cells (WI-38 sen) are still able to mount a DDR as detected in the form of γH2AX foci 1 day after induction of DNA damage by irradiation with 20 Gy, similarly to BJ senescent cells (BJ sen). Scale bar, 5 µm. (PPTX) [file pone.0110969.s006.pptx]

## Slide 1
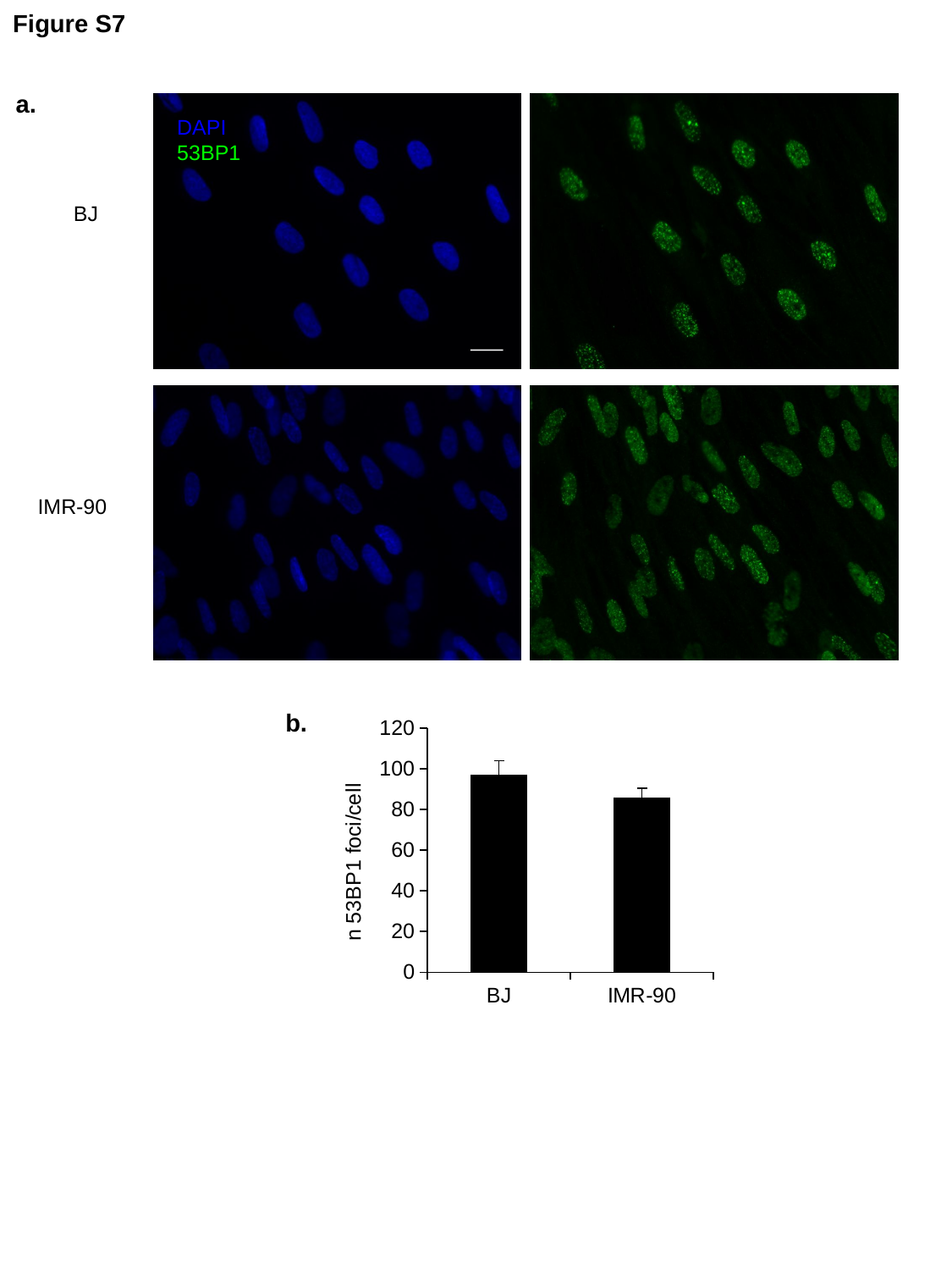

Figure S7
a.
DAPI
53BP1
BJ
IMR-90
b.
### Chart
| Category |
|---|
### Chart
| Category | |
|---|---|
| BJ | 97.12765957446808 |
| IMR-90 | 85.63636363636364 |

Supplement: Figure S7 — BJ and IMR-90 activate a comparable DDR initially upon irradiation. BJ and IMR-90 cells were irradiated with 1 Gy and stained 10 minutes later. a. Representative images of 53BP1 foci. Scale bar, 20 µm. b. Quantification of number of 53BP1 foci per cell. (PPTX) [file pone.0110969.s007.pptx]

## Slide 1
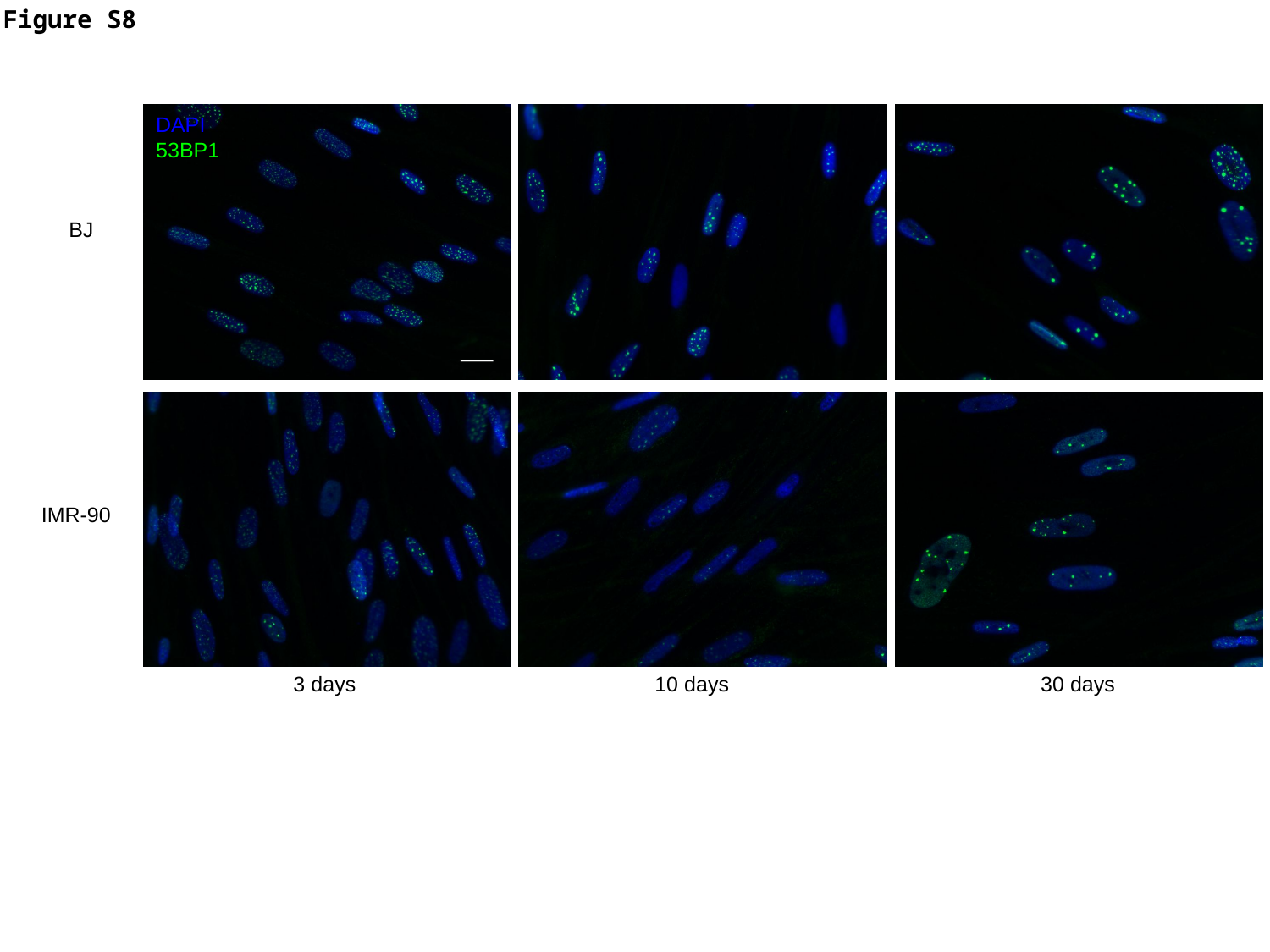

Figure S8
DAPI
53BP1
BJ
IMR-90
3 days
10 days
30 days

Supplement: Figure S8 — Differential kinetics of DDR foci resolution in different cell types. Representative pictures of 53BP1 foci in BJ and IMR-90 at the indicated time points after 20 Gy irradiation. Scale bar, 20 µm. (PPTX) [file pone.0110969.s008.pptx]

## Slide 1
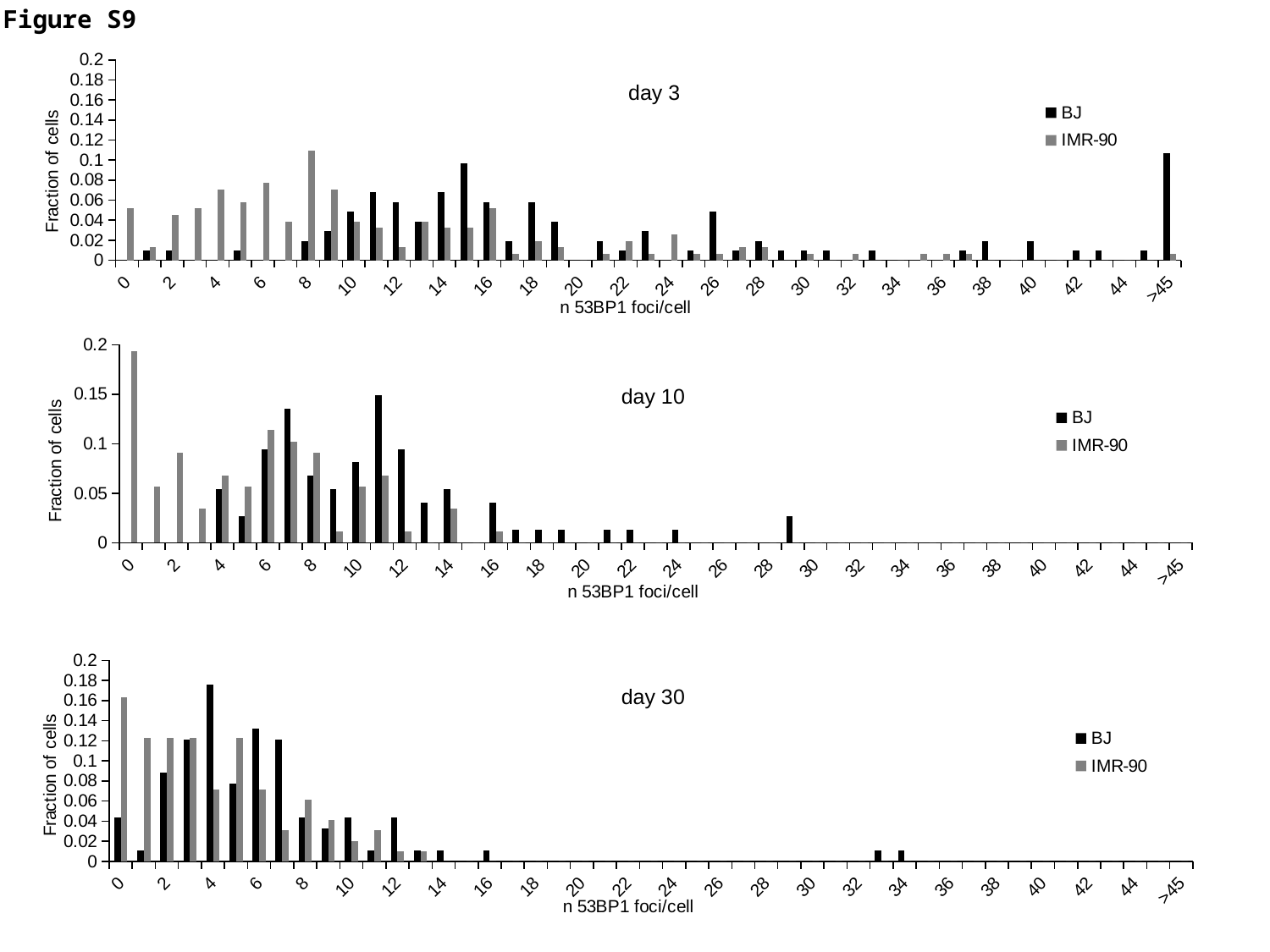

Figure S9
### Chart
| Category | BJ | IMR-90 |
|---|---|---|
| 0 | 0.0 | 0.0516129032258064 |
| 1 | 0.00970873786407767 | 0.0129032258064516 |
| 2 | 0.00970873786407767 | 0.0451612903225806 |
| 3 | 0.0 | 0.0516129032258064 |
| 4 | 0.0 | 0.0709677419354839 |
| 5 | 0.00970873786407767 | 0.0580645161290322 |
| 6 | 0.0 | 0.0774193548387097 |
| 7 | 0.0 | 0.0387096774193548 |
| 8 | 0.0194174757281553 | 0.109677419354839 |
| 9 | 0.029126213592233 | 0.0709677419354839 |
| 10 | 0.0485436893203883 | 0.0387096774193548 |
| 11 | 0.0679611650485437 | 0.032258064516129 |
| 12 | 0.058252427184466 | 0.0129032258064516 |
| 13 | 0.0388349514563107 | 0.0387096774193548 |
| 14 | 0.0679611650485437 | 0.032258064516129 |
| 15 | 0.0970873786407767 | 0.032258064516129 |
| 16 | 0.058252427184466 | 0.0516129032258064 |
| 17 | 0.0194174757281553 | 0.0064516129032258 |
| 18 | 0.058252427184466 | 0.0193548387096774 |
| 19 | 0.0388349514563107 | 0.0129032258064516 |
| 20 | 0.0 | 0.0 |
| 21 | 0.0194174757281553 | 0.0064516129032258 |
| 22 | 0.00970873786407767 | 0.0193548387096774 |
| 23 | 0.029126213592233 | 0.0064516129032258 |
| 24 | 0.0 | 0.0258064516129032 |
| 25 | 0.00970873786407767 | 0.0064516129032258 |
| 26 | 0.0485436893203883 | 0.0064516129032258 |
| 27 | 0.00970873786407767 | 0.0129032258064516 |
| 28 | 0.0194174757281553 | 0.0129032258064516 |
| 29 | 0.00970873786407767 | 0.0 |
| 30 | 0.00970873786407767 | 0.0064516129032258 |
| 31 | 0.00970873786407767 | 0.0 |
| 32 | 0.0 | 0.0064516129032258 |
| 33 | 0.00970873786407767 | 0.0 |
| 34 | 0.0 | 0.0 |
| 35 | 0.0 | 0.0064516129032258 |
| 36 | 0.0 | 0.0064516129032258 |
| 37 | 0.00970873786407767 | 0.0064516129032258 |
| 38 | 0.0194174757281553 | 0.0 |
| 39 | 0.0 | 0.0 |
| 40 | 0.0194174757281553 | 0.0 |
| 41 | 0.0 | 0.0 |
| 42 | 0.00970873786407767 | 0.0 |
| 43 | 0.00970873786407767 | 0.0 |
| 44 | 0.0 | 0.0 |
| 45 | 0.00970873786407767 | 0.0 |
| >45 | 0.106796116504854 | 0.0064516129032258 |day 3
### Chart
| Category | BJ | IMR-90 |
|---|---|---|
| 0 | 0.0 | 0.193181818181818 |
| 1 | 0.0 | 0.0568181818181818 |
| 2 | 0.0 | 0.0909090909090909 |
| 3 | 0.0 | 0.0340909090909091 |
| 4 | 0.054054054054054 | 0.0681818181818182 |
| 5 | 0.027027027027027 | 0.0568181818181818 |
| 6 | 0.0945945945945946 | 0.113636363636364 |
| 7 | 0.135135135135135 | 0.102272727272727 |
| 8 | 0.0675675675675676 | 0.0909090909090909 |
| 9 | 0.054054054054054 | 0.0113636363636364 |
| 10 | 0.0810810810810811 | 0.0568181818181818 |
| 11 | 0.148648648648649 | 0.0681818181818182 |
| 12 | 0.0945945945945946 | 0.0113636363636364 |
| 13 | 0.0405405405405405 | 0.0 |
| 14 | 0.054054054054054 | 0.0340909090909091 |
| 15 | 0.0 | 0.0 |
| 16 | 0.0405405405405405 | 0.0113636363636364 |
| 17 | 0.0135135135135135 | 0.0 |
| 18 | 0.0135135135135135 | 0.0 |
| 19 | 0.0135135135135135 | 0.0 |
| 20 | 0.0 | 0.0 |
| 21 | 0.0135135135135135 | 0.0 |
| 22 | 0.0135135135135135 | 0.0 |
| 23 | 0.0 | 0.0 |
| 24 | 0.0135135135135135 | 0.0 |
| 25 | 0.0 | 0.0 |
| 26 | 0.0 | 0.0 |
| 27 | 0.0 | 0.0 |
| 28 | 0.0 | 0.0 |
| 29 | 0.027027027027027 | 0.0 |
| 30 | 0.0 | 0.0 |
| 31 | 0.0 | 0.0 |
| 32 | 0.0 | 0.0 |
| 33 | 0.0 | 0.0 |
| 34 | 0.0 | 0.0 |
| 35 | 0.0 | 0.0 |
| 36 | 0.0 | 0.0 |
| 37 | 0.0 | 0.0 |
| 38 | 0.0 | 0.0 |
| 39 | 0.0 | 0.0 |
| 40 | 0.0 | 0.0 |
| 41 | 0.0 | 0.0 |
| 42 | 0.0 | 0.0 |
| 43 | 0.0 | 0.0 |
| 44 | 0.0 | 0.0 |
| 45 | 0.0 | 0.0 |
| >45 | 0.0 | 0.0 |day 10
### Chart
| Category | BJ | IMR-90 |
|---|---|---|
| 0 | 0.043956043956044 | 0.163265306122449 |
| 1 | 0.010989010989011 | 0.122448979591837 |
| 2 | 0.0879120879120879 | 0.122448979591837 |
| 3 | 0.120879120879121 | 0.122448979591837 |
| 4 | 0.175824175824176 | 0.0714285714285714 |
| 5 | 0.0769230769230769 | 0.122448979591837 |
| 6 | 0.131868131868132 | 0.0714285714285714 |
| 7 | 0.120879120879121 | 0.0306122448979592 |
| 8 | 0.043956043956044 | 0.0612244897959184 |
| 9 | 0.032967032967033 | 0.0408163265306122 |
| 10 | 0.043956043956044 | 0.0204081632653061 |
| 11 | 0.010989010989011 | 0.0306122448979592 |
| 12 | 0.043956043956044 | 0.0102040816326531 |
| 13 | 0.010989010989011 | 0.0102040816326531 |
| 14 | 0.010989010989011 | 0.0 |
| 15 | 0.0 | 0.0 |
| 16 | 0.010989010989011 | 0.0 |
| 17 | 0.0 | 0.0 |
| 18 | 0.0 | 0.0 |
| 19 | 0.0 | 0.0 |
| 20 | 0.0 | 0.0 |
| 21 | 0.0 | 0.0 |
| 22 | 0.0 | 0.0 |
| 23 | 0.0 | 0.0 |
| 24 | 0.0 | 0.0 |
| 25 | 0.0 | 0.0 |
| 26 | 0.0 | 0.0 |
| 27 | 0.0 | 0.0 |
| 28 | 0.0 | 0.0 |
| 29 | 0.0 | 0.0 |
| 30 | 0.0 | 0.0 |
| 31 | 0.0 | 0.0 |
| 32 | 0.0 | 0.0 |
| 33 | 0.010989010989011 | 0.0 |
| 34 | 0.010989010989011 | 0.0 |
| 35 | 0.0 | 0.0 |
| 36 | 0.0 | 0.0 |
| 37 | 0.0 | 0.0 |
| 38 | 0.0 | 0.0 |
| 39 | 0.0 | 0.0 |
| 40 | 0.0 | 0.0 |
| 41 | 0.0 | 0.0 |
| 42 | 0.0 | 0.0 |
| 43 | 0.0 | 0.0 |
| 44 | 0.0 | 0.0 |
| 45 | 0.0 | 0.0 |
| >45 | 0.0 | 0.0 |day 30

Supplement: Figure S9 — DDR foci distribution in BJ and IMR-90 at different time points following irradiation. Histograms show the distribution of 53BP1 foci for data in Fig. 4b. (PPTX) [file pone.0110969.s009.pptx]
