# Supplementary material for: Stable Cellular Senescence Is Associated with Persistent DDR Activation
Source: PLoS One. 2014 Oct 23;9(10):e110969. doi: 10.1371/journal.pone.0110969 (PMC4207795; doi:10.1371/journal.pone.0110969)
Supplement: Figure S3 — Senescence establishment in WI-38 and BJ fibroblasts detected by SA-β-gal staining and BrdU incorporation assay. WI-38 and BJ senescent cells (WI-38 sen and BJ sen respectively) show low level of BrdU incorporation (24 hours pulse) compared to the pre-senescent (pre-sen) and proliferating ones (prol) and an increase in SA-β-gal activity. Error bars represent s.e.m. (PPTX) [file pone.0110969.s003.pptx]

## Slide 1
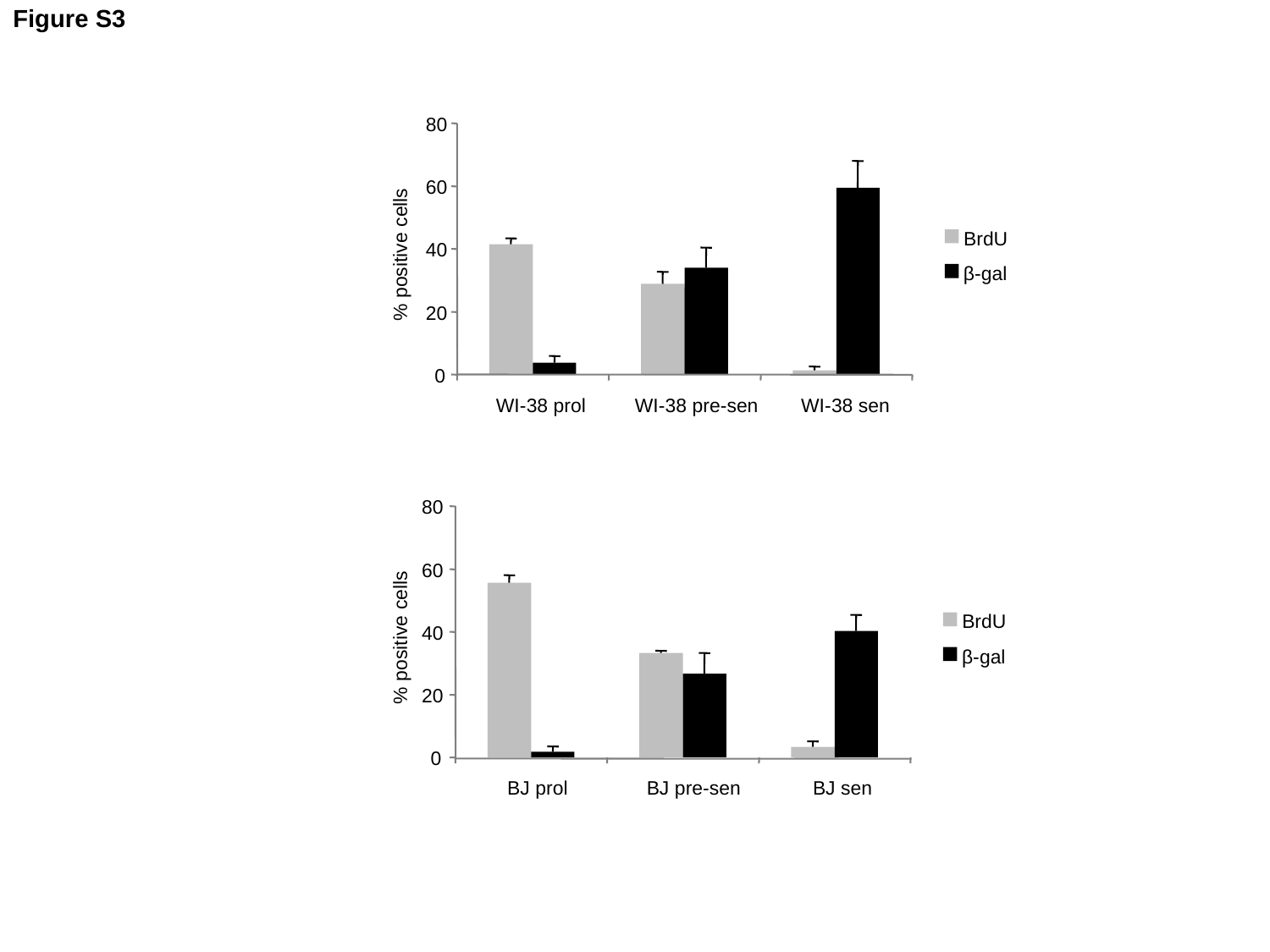

Figure S3
80
60
BrdU
% positive cells
40
β-gal
20
0
WI-38 prol
WI-38 pre-sen
WI-38 sen
80
60
BrdU
% positive cells
40
β-gal
20
0
BJ prol
BJ pre-sen
BJ sen
