# Supplementary material for: Stable Cellular Senescence Is Associated with Persistent DDR Activation
Source: PLoS One. 2014 Oct 23;9(10):e110969. doi: 10.1371/journal.pone.0110969 (PMC4207795; doi:10.1371/journal.pone.0110969)
Supplement: Figure S5 — DDR foci distribution in BJ and WI-38 before and at different time points after senescence establishment. Histograms show the distribution of γH2AX foci for data in Fig. 3a. (PPTX) [file pone.0110969.s005.pptx]

## Slide 1
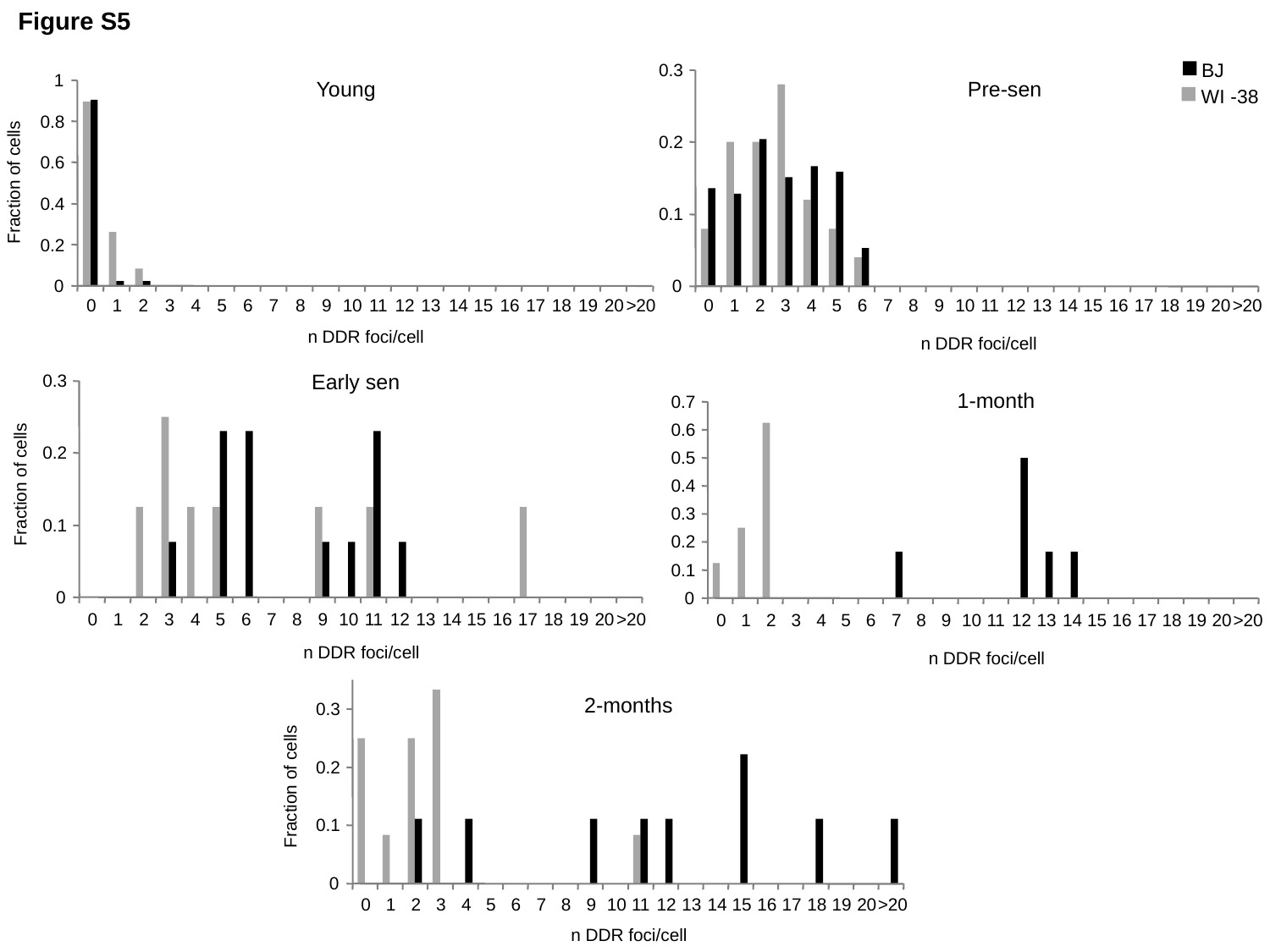

Figure S5
BJ
0.3
1
Young
Pre-sen
WI -38
0.8
0.2
0.6
Fraction of cells
0.4
0.1
0.2
0
0
0
1
2
3
4
5
6
7
8
9
10
11
12
13
14
15
16
17
18
19
20
>20
0
1
2
3
4
5
6
7
8
9
10
11
12
13
14
15
16
17
18
19
20
>20
n DDR foci/cell
n DDR foci/cell
Early sen
0.3
1-month
0.7
0.6
0.2
0.5
Fraction of cells
0.4
0.3
0.1
0.2
0.1
0
0
0
1
2
3
4
5
6
7
8
9
10
11
12
13
14
15
16
17
18
19
20
>20
0
1
2
3
4
5
6
7
8
9
10
11
12
13
14
15
16
17
18
19
20
>20
n DDR foci/cell
n DDR foci/cell
2-months
0.3
0.2
Fraction of cells
0.1
0
0
1
2
3
4
5
6
7
8
9
10
11
12
13
14
15
16
17
18
19
20
>20
n DDR foci/cell
